# Quantitative Imaging Advances in HPV-Positive Oropharyngeal Carcinoma †

**DOI:** 10.3390/cancers18020303

**Published:** 2026-01-19

**Authors:** Dermot Farrell, Houda Bahig, Richard Khor, Luiz P. Kowalski, Remco de Bree, Avraham Eisbruch, Heleen Bollen, Fernando Lopez, M. P. Sreeram, Orlando Guntinas-Lichius, Juan P. Rodrigo, Nabil F. Saba, Karthik N. Rao, Sandra Nuyts, Anna Luíza Damaceno Araújo, Alfio Ferlito, Sweet Ping Ng

**Affiliations:** 1 Department of Radiation Oncology, Austin Health, Melbourne, VIC 3084, Australiarichard.khor@austin.org.au (R.K.); 2 Department of Radiation Oncology, Centre Hospitalier de l’Universite de Montreal, Montreal, QC H2X 0C1, Canada; houda.bahig.med@ssss.gouv.qc.ca; 3 Department of Head and Neck Surgery and Otorhinolaryngology, A.C. Camargo Cancer Center, São Paulo 01509-010, Brazil; luiz.kowalski@hc.fm.usp.br; 4 Head and Neck Surgery Department and LIM 28, University of São Paulo Medical School and Hospital Israelita Albert Einstein, São Paulo 05508-020, Brazil; anna_luizaf5ph@hotmail.com; 5 Department of Head and Neck Surgical Oncology, University Medical Center Utrecht, 3508 GA Utrecht, The Netherlands; r.debree@umcutrecht.nl; 6 Department of Radiation Oncology, University of Michigan, Ann Arbor, MI 48109, USA; eisbruch@med.umich.edu; 7 Laboratory of Experimental Radiotherapy, Department of Radiation Oncology, Leuven Cancer Institute, University Hospitals Leuven, 3000 Leuven, Belgium; heleen.bollen@uzleuven.be; 8 ENT and Head and Neck Department, Hospital Universitario Central de Asturias, 33011 Oviedo, Spain; lopezafernando@uniovi.es; 9 Department of Head and Neck Oncology, Sri Shankara Cancer Hospital and Research Centre, Bangalore 560004, India; sreeram.mp@sschrc.org (M.P.S.); karthikrao@sschrc.org (K.N.R.); 10 Department of Otorhinolaryngology, Jena University Hospital, Am Klinikum 1, D-07747 Jena, Germany; orlando.guntinas@med.uni-jena.de; 11 Department of Otolaryngology, Hospital Universitario Central de Asturias and Instituto de Investigación Sanitaria del Principado de Asturias (ISPA), Instituto Universitario de Oncología del Principado de Asturias, University of Oviedo, 33011 Oviedo, Spain; jprodrigo@uniovi.es; 12 Department of Hematology and Medical Oncology, Winship Cancer Institute, Emory University, Atlanta, GA 30322, USA; nfsaba@emory.edu; 13 Department of Radiation Oncology, University Hospitals Leuven, KU Leuven-University of Leuven, 3000 Leuven, Belgium; sandra.nuyts@uzleuven.be; 14 Hospital Israelita Albert Einstein, São Paulo 05652-900, Brazil; 15 International Head and Neck Scientific Group, 35100 Padua, Italy; profalfioferlito@gmail.com

**Keywords:** HPV, oropharyngeal, MRI, CT, PET, radiomics

## Abstract

HPV-positive OPSCC shows a favourable prognosis, prompting evaluation of de-escalated and adaptive strategies. Quantitative imaging may provide scalable biomarkers to individualise care. Quantitative imaging can support baseline risk stratification, early on-treatment decision-making, and posttreatment surveillance in HPV-positive OPSCC. Real-world translation requires standardised reporting, calibration/harmonisation across centres, rigorous model validation, and workflow integration with radiotherapy planning. Quantitative MRI, CT, and PET, augmented by radiomics and AI, show convergent promise as non-invasive biomarkers to enable safe individualisation of therapy in HPV-positive OPSCC, contingent on methodological rigour and prospective, externally validated studies. Despite this promise, clinical translation faces substantial barriers, including limited external validation, heterogeneous methodologies, and the need for standardised, prospectively validated pipelines.

## Simple Summary

This is a review article of published research on the area of quantitative imaging for HPV-positive oropharyngeal carcinomas. This review looks at advances in multiple modalities, including MRI, CT, PET, and the use of deep learning tools in this field. Progress in this field will potentially change diagnostic workflows for relevant patients. Unlike prior narrative reviews that have primarily catalogued modality-specific performance, this review focuses on both decision and implementation by mapping results from imaging investigations to decision points found in the radiotherapy pathway (diagnosis, treatment adaptation, and post-treatment surveillance). Further, we have highlighted where evidence is now informing de-escalation vs. where evidence still remains exploratory. We have summarised technical and validation requirements for embedding these biomarkers into radiotherapy workflows and de-escalation trials.

## 1. Introduction

Human papillomavirus (HPV)-positive oropharyngeal squamous cell carcinoma (OPSCC) is biologically distinct from HPV-negative disease and generally carries a more favourable prognosis [1]. This survival advantage has fuelled interest in treatment de-escalation strategies; however, safely individualising therapy depends on robust biomarkers that can identify risk, forecast response early, and guide posttreatment decisions. Quantitative imaging is well positioned to meet this need because it can be acquired non-invasively at scale, repeated longitudinally, and integrated with radiotherapy planning.

Despite the favourable prognosis of HPV-positive OPSCC, clinically meaningful heterogeneity persists: a subset of patients experiences recurrence or treatment-related morbidity that is not well predicted by current staging and clinicopathologic stratification alone. This creates three practical gaps that motivate this review. Firstly, risk categorisation remains coarse—clinicians cannot reliably distinguish those suitable for de-escalation from those who require standard (or intensified) therapy using routinely available variables, particularly when competing objectives include cure, function preservation, and long-term toxicity [2]. Secondly, treatment modification is limited by the lack of validated early-response triggers—during radiotherapy (± systemic therapy), adaptation decisions are often constrained to anatomy- or symptom-driven changes, while biological response may precede visible size change and is rarely quantified in a standardised way [3,4]. Thirdly, posttreatment monitoring is frequently uncertain—equivocal findings can lead to additional imaging, invasive procedures, or delayed salvage, highlighting the need for objective criteria that better triage patients after treatment [5]. Imaging biomarkers are anticipated to address these gaps by providing scalable, longitudinal, non-invasive measurements that can support decision-making at diagnosis, during treatment, and in surveillance—provided they are technically robust and prospectively validated.

Contemporary radiotherapy pathways already rely on multimodality imaging for staging, target delineation, and response assessment. Embedding quantitative readouts within these offers a pragmatic route to precision care: baseline models can refine risk at diagnosis; on-treatment metrics can provide early readouts to trigger adaptive strategies; and posttreatment endpoints can standardise surveillance and triage. Importantly, quantitative methods bring the possibility of objective, reproducible measurements across centres—an important property if predictors are to support de-intensification without compromising disease control.

Quantitative imaging in head and neck oncology is evolving rapidly beyond conventional radiology, driven by advances in machine learning and new sensing modalities. For example, hyperspectral imaging combined with computer-aided diagnostic methods is increasingly being explored to enhance detection and diagnosis in head and neck cancer, reflecting a broader shift towards high-dimensional, algorithm-assisted tissue characterisation [6].

In parallel, contemporary reviews of AI-driven radiomics in head and neck cancer highlight both the breadth of emerging applications and persistent translation barriers—particularly limited external validation, site/scanner variability, and practical workflow integration [7]. Against this backdrop, the key unmet need is not another modality-specific catalogue of model performance but a clinically anchored synthesis focused on HPV-positive OPSCC that maps quantitative imaging biomarkers to real decision points (risk stratification for de-escalation, response-adaptive treatment modification, and posttreatment assessment) while critically evaluating methodological robustness and readiness for multi-centre testing and implementation.

Translation hinges on technical robustness as much as on statistical significance. Feature values vary with acquisition, reconstruction, segmentation policy, and preprocessing; consequently, adherence to consensus definitions and transparent reporting (e.g., exact b-values for diffusion, reconstruction kernels for CT, and response criteria for PET) is central to external validity. Throughout this review, we highlight where the biomarker signal is most consistent, where it attenuates after appropriate adjustment (e.g., for HPV status and stage), and how study design choices (class imbalance handling, leakage-free validation, and multi-centre testing) influence apparent performance.

This review synthesises advances across four complementary pillars:

1. **Diffusion-weighted MRI (DWI) and apparent diffusion coefficient (ADC) mapping**—capturing tumour microstructure and early therapy-induced change. Multiple OPSCC cohorts report lower baseline ADC in HPV-positive tumours and robust **midtreatment ΔADC (change in ADC)** signals that anticipate outcomes [8,9], whereas baseline associations can attenuate after adjusting for HPV status and stage [8,10]. Prospective studies and MR-Linac feasibility work highlight clinically actionable timepoints for adaptive strategies [11,12].
2. **MRI radiomics**—converting routine sequences (contrast-enhanced T1 weighted imaging (CE-T1), T2, and ADC) into high-throughput descriptors of phenotype. Across single-centre and multi-centre analyses, diffusion-derived features frequently rank among the most informative for **HPV/p16 classification** [13], and radiomics **augments** clinicopathologic models for survival rather than replacing them. Methodological appraisals underline the importance of class-imbalance handling, feature stability, and transparent validation [14,15].
3. **CT radiomics and deep learning**—leveraging ubiquitous planning CT. Early texture studies showed reproducible **HPV-related signals** on CT [16], subsequently validated across centres; nodal features add complementary information to primary tumour features [3]. CT radiomics improves overall survival/progression-free survival **(OS/PFS) and locoregional-control** prediction when combined with clinical variables, while modern deep learning approaches demonstrate segmentation-free HPV classification and improved extranodal-extension screening on trial data.
4. **FDG-PET/CT metrics and radiomics**—quantifying metabolic burden and heterogeneity. Volumetric indices such as **metabolic tumour volume (MTV)** and **total lesion glycolysis (TLG)** outperform single-voxel standardised uptake value (SUV) measures for prognosis, including within HPV-positive cohorts. Texture features add signals beyond MTV/TLG, and **PERCIST-aligned** longitudinal endpoints [17] plus **PET-NECK** trial evidence support [5] PET-guided post-chemoradiotherapy (CRT) management.

## 2. Materials and Methods

### 2.1. Review Design

This manuscript is a structured narrative review of quantitative imaging biomarkers in HPV-positive oropharyngeal squamous cell carcinoma (OPSCC), focused on (i) diffusion-weighted MRI (DWI)/apparent diffusion coefficient (ADC) and diffusion radiomics; (ii) radiomics and machine learning/deep learning models across MRI/CT/PET; (iii) PET-based metrics and response frameworks; and (iv) translational readiness, including workflow integration and external validation. The purpose was to synthesise evidence around clinically relevant decision points (baseline risk stratification and de-escalation selection; on-treatment response assessment for adaptive strategies; posttreatment assessment/surveillance).

### 2.2. Information Sources

A literature search was conducted on PubMed/MEDLINE, Embase, Scopus, Web of Science Core Collection, and the Cochrane Library. Searches were supplemented by backward citation searching (screening reference lists of key included papers and relevant reviews) and forward citation searching (“cited by” functions in Scopus/Web of Science).

### 2.3. Search Strategy and Limits

Searches combined three concept groups:

1. Disease/site: Oropharyngeal cancer, OPSCC, oropharyngeal squamous cell carcinoma, head and neck squamous cell carcinoma.
2. HPV status: HPV, p16, human papillomavirus.
3. Quantitative imaging/computational methods: Diffusion-weighted imaging (DWI), ADC/apparent diffusion coefficient, IVIM; radiomics/texture analysis; machine learning, deep learning, artificial intelligence; CT, MRI, FDG-PET/PET-CT; MR-Linac/MR-guided radiotherapy and adaptive radiotherapy; and methodological terms, including standardisation, reproducibility/repeatability, harmonisation (including ComBat), multi-centre design, and external validation.

Limits applied: January 2009 to June 2025, English language, and human studies. An example full database search strategy is provided in Appendix A. When restricted to 1 January 2009–31 June 2025, PubMed keyword searches illustrate both the scale and fragmentation of the literature that this review synthesises: HPV (46,775 records), oropharyngeal (28,583), MRI (614,877), CT (510,021), PET (133,252), and radiomics (17,570).

### 2.4. Eligibility Criteria

Included studies were peer-reviewed journal publications relevant to HPV-positive OPSCC and quantitative imaging, including (i) original clinical studies (prospective or retrospective); (ii) multi-centre or externally validated studies where available; (iii) trial-embedded imaging analyses; and (iv) high-quality systematic reviews, guidelines, and standards, directly informing quantitative imaging methodology or response assessment.

Excluded sources were preprints, conference abstracts without full manuscripts, theses, non-indexed reports, and other non-peer-reviewed materials. Studies of mixed head and neck cohorts were included only when HPV-positive OPSCC data were reported separately or constituted a major analytic focus.

Generative AI was used in the writing of this review article. Portions of this manuscript were edited using an AI-assisted language tool (ChatGPT, OpenAI, https://chat.openai.com, accessed on 16 January 2026) to improve clarity, grammar, and flow. All scientific content, interpretations, and conclusions are those of the authors, who reviewed and approved the final text.

## 3. Results

### 3.1. Diffusion-Weighted MRI (DWI) and Apparent Diffusion Coefficient (ADC) Mapping

Diffusion-weighted MRI (DWI) is a functional MRI technique that sensitises the signal to the random (Brownian) motion of water molecules within tissue. The derived apparent diffusion coefficient (ADC) quantifies this motion and provides a surrogate measure of tumour cellularity, stromal architecture, and necrosis. In solid tumours, highly cellular regions with intact cell membranes typically exhibit restricted diffusion and lower ADC, whereas treatment-induced cell death, oedema, and necrosis lead to less restricted diffusion and higher ADC. Because of this, DWI and ADC offer a non-invasive way to probe tumour microstructure beyond what is visible on conventional anatomical MRI. They are particularly attractive as imaging biomarkers because they can be acquired on most modern clinical MRI systems without contrast, are relatively quick to perform, and can be repeated throughout the course of chemoradiotherapy to track early biological response and microstructural change.

#### 3.1.1. Baseline ADC and HPV Phenotyping

Across key diffusion studies in head and neck squamous cell carcinoma, including OPSCC, DWI/ADC has been evaluated at three clinically relevant timepoints: at baseline, during chemoradiotherapy (CRT), and early after CRT. In a prospective HNSCC cohort with mixed subsites, Vandecaveye et al. acquired DWI at baseline and again at weeks 2 and 4 of CRT, showing that serial ΔADC increases were significantly larger in complete responders than in non-responders, with early ΔADC correlating with 2-year local control [11]. A subsequent prospective study from the same group obtained DWI 2–4 weeks after CRT and found that early posttreatment ADC had a very high negative predictive value for residual primary tumours (negative predictive value ≈ 100% at the primary site and ≈ 91% for nodal disease), supporting DWI as a triage tool to avoid unnecessary early salvage surgery or biopsy [18]. In a complementary baseline-focused analysis, Ravanelli et al. studied advanced OPSCC with known HPV status and showed that pretreatment ADC histogram metrics were associated with progression-free and overall survival, with heterogeneity measures such as entropy predicting overall survival in HPV-negative disease [19]. Together, these findings illustrate why DWI/ADC is emphasised in this review: it provides repeatable, non-invasive readouts that capture early microstructural response and baseline heterogeneity, and it has demonstrated associations with control, survival, and the need for salvage interventions across HNSCC and HPV-aware OPSCC cohorts [11,18,19].

#### 3.1.2. Baseline ADC as a Prognostic Marker

Multiple OPSCC cohorts report lower baseline ADC—that is, pretreatment ADC metrics derived from MRI acquired before chemoradiotherapy—in HPV-positive primary tumours, with additional discriminative value from ADC histogram and texture features. In a focused diffusion study of 34 patients, Lenoir et al. acquired 3T DWI with six b-values (0–1000 s/mm^2^), reconstructed multiple ADC maps, and showed that the ability of ADC histograms to distinguish HPV status depended strongly on the chosen b-value combination; kurtosis on the ADC_{b0–1000} map achieved the best separation of HPV-positive from HPV-negative OPSCC (AUC ≈ 0.89, sensitivity: 100%, and specificity: 82.6%) [20]. Ravanelli and colleagues similarly demonstrated that pretreatment mean ADC was significantly lower in HPV-positive OPSCC and that ADC histogram parameters related to progression-free and overall survival in advanced disease with known HPV status [9,19]. In a hybrid PET/MRI cohort, Freihat et al. also found lower pretreatment primary tumour ADC_{mean} in HPV-positive patients and reported that ADC_{mean}, but not FDG-PET metrics, helped predict chemoradiotherapy response [21]. However, this pattern is not universal. In a 44-patient OPSCC series with p16-based HPV testing, Schouten et al. observed no statistically significant difference in baseline mean ADC between HPV-positive and HPV-negative tumours [8], underscoring the influence of acquisition parameters, ROI definition, and cohort composition on reported ADC–HPV associations.

Early diffusion studies reported an association between lower pretreatment ADC and subsequent local treatment failure after radiotherapy or chemoradiotherapy [12]. These were typically retrospective, single-centre head and neck cohorts that included mixed subsites (often not limited to OPSCC), used variable DWI protocols (different b-value schemes and readouts), and lacked systematic HPV stratification. Patients who later failed treatment tended to show lower baseline ADC at diagnosis, which was interpreted as reflecting higher cellularity and more restricted diffusion in biologically aggressive tumours. However, these analyses were constrained by small sample sizes, selection bias, heterogeneous acquisition and segmentation methods, and—critically—the absence of adjustment for HPV status and stage, both of which are major prognostic determinants in OPSCC. Consequently, the observed association between low baseline ADC and failure may reflect underlying tumour biology and protocol-related artefacts as much as any independent prognostic contribution from ADC itself [12].

Across key diffusion studies in head and neck squamous cell carcinoma, a consistent pattern emerges. Baseline ADC and ADC-histogram metrics often correlate with HPV status and outcomes, but their independent prognostic value tends to attenuate once HPV/p16 status, T/N stage, and other clinical factors are included, and effect sizes are sensitive to b-value schemes, ROI definition, and segmentation. In contrast, midtreatment ΔADC—typically measured at week 2–4 of CRT—shows more robust and reproducible associations with locoregional control and survival, with low early ΔADC repeatedly identifying poor-responding tumours that are candidates for treatment intensification or early salvage [8,9,11,18,19,20,21,22].

Across studies, baseline ADC sometimes appears prognostic, but the direction and magnitude of associations are not uniform and, in several analyses, weaken when models account for dominant clinical determinants such as HPV status, stage, and related covariates [10,22]. This pattern suggests that part of the apparent prognostic signal may reflect correlated biology (e.g., HPV-linked tumour microstructure) or protocol-dependent measurement differences rather than an independent risk factor [8,20]. Heterogeneity in acquisition (b-value scheme, fitting model, distortion correction), segmentation definitions (primary vs. nodal; whole lesion vs. solid tumour; necrosis/cyst handling), and outcome definitions further shifts ADC distributions and cut-points, plausibly explaining why similar cohorts can yield different “optimal” thresholds and performance [14,20]. Clinically, baseline ADC should, therefore, be interpreted as a candidate adjunct that requires demonstration of incremental value (calibration/reclassification/decision benefit) beyond standard predictors, particularly within HPV-positive cohorts.

#### 3.1.3. Methodological Considerations and Link to MRI Radiomics

MR-Linac implementations further demonstrate that weekly on-treatment DWI is both technically feasible and biologically informative. In a prospective R-IDEAL stage 2a study on a 1.5 T MR-Linac, thirty HNSCC patients underwent baseline and weekly DWI during radiotherapy; ADC metrics rose significantly over the course of treatment for both gross primary volume (GTV-P) and nodal disease (GTV-N), with larger mid-treatment ΔADC in primaries achieving complete remission [23]. Recursive partitioning identified a GTV-P ΔADC 5th percentile > 13% at mid-RT as the strongest discriminator of complete response, and increasing ADC was associated with progressive reductions in residual tumour volume and a negative correlation between ΔADC and change in volume [23]. These data confirm the feasibility of serial intra-treatment DWI on MR-Linac platforms and support the concept of ΔADC-guided, response-adapted planning within the same treatment course [23].

Complementing this, multiparametric MRI radiomics that include ADC features have repeatedly surfaced diffusion-derived descriptors among the most informative predictors, and cross-modal PET/MRI work has shown concordant pretreatment differences—with ADC relating to HPV status/response, while FDG-PET metrics (e.g., MTV, TLG) contribute orthogonal biological information about tumour metabolism. Together, these findings suggest that ADC-based histogram/texture features add complementary signals within multi-sequence MRI and PET/MRI models and can strengthen phenotype or response prediction when diffusion acquisition and feature extraction are standardised [21].

Against this background, we must also consider baseline diffusion signatures that differentiate HPV phenotypes. Head and neck DWI is highly sensitive to motion, susceptibility, and protocol heterogeneity, including field strength, echo-planar readout, fat suppression, and b-value selection. Lower b-values increase perfusion sensitivity, whereas very high b-values reduce signal-to-noise ratios; both effects alter ADC histogram shape and the stability of texture features. In an OPSCC cohort, Lenoir et al. systematically varied b-value combinations and showed that ADC kurtosis on an ADC_{b0–1000} map best distinguished HPV-positive from HPV-negative tumours, with performance dropping when only higher b-pairs were used [20]. By contrast, Schouten et al. used mean ADC from single-slice ROIs with a different b-value scheme and found no significant baseline ADC difference by HPV [8]. Together, these data illustrate how motion control, readout/fat suppression, segmentation policy, and b-value schemes can drive conflicting conclusions about HPV separability, underscoring the need for explicit reporting and standardisation when interpreting ADC-based phenotyping in OPSCC [8,20].

To improve generalisability, feature extraction should be aligned with the Image Biomarker Standardisation Initiative (IBSI) and reported in a way that allows for full reproducibility. In practice, this means, at minimum, (i) reporting exact b-values and diffusion readouts (including EPI and fat-suppression choices, which directly affect diffusion contrast and derived metrics), (ii) stating the diffusion model used for ADC (mono-exponential vs. alternatives) and key fitting options (e.g., noise-floor handling), (iii) defining the segmentation policy (2D vs. 3D; manual vs. semi-automatic; software/tool; thresholds and morphological edits) and how inter-observer variability was managed (consensus contours, repeated segmentations, or agreement statistics), and (iv) specifying the timing and calculation of ΔADC (week 2/week 4 or other prespecified timepoints; absolute vs. percentage change; whole-lesion vs. subvolume; per-patient aggregation rules). Systematic methodological reviews highlight inconsistent acquisition, segmentation, and feature protocols as major sources of between-study variability and modest Radiomics Quality Scores, underlining the need for standardised pipelines [24]. IBSI provides consensus feature definitions, reference values, and reporting checklists that improve cross-software agreement and test–retest reliability for DWI and radiomics workflows [14]. In parallel, HPV-aware ADC studies show that ostensibly strong prognostic effects can disappear once p16 status and clinical covariates are properly modelled [10], reinforcing the need to document both imaging methodology and statistical adjustment. Accordingly, minimum reportable items include scanner/vendor and field strength; sequence type and bandwidth; all diffusion b-values; motion and fat-suppression strategy; ADC model and fitting details; ROI workflow and quality control; ΔADC definition and normalisation; and any preprocessing (registration, resampling, intensity normalisation/clipping) prior to feature extraction—each directly affecting reproducibility and comparability across centres [10,14,24].

For HPV-associated OPSCC, midtreatment ΔADC demonstrates the most consistent link to outcomes: across prospective cohorts with prespecified week-2/week-4 DWI, patients who ultimately achieve better locoregional control show larger intratreatment ΔADC, and this effect persists in multivariable analyses that include HPV status and stage [11,18,22]. MR-Linac programmes further show that on-treatment DWI is feasible on a weekly basis, with systematic midtreatment ADC rises in primary and nodal disease, supporting the clinical concept of response-adapted management during the same course of CRT [23].

Baseline ADC and ADC-radiomics assist HPV phenotyping and can augment risk models when acquisition is controlled: studies using standardised b-value schemes and consistent ROI/feature extraction report that pretreatment ADC distributions and histogram shape (e.g., kurtosis/skewness) help distinguish HPV phenotypes and relate to early responses [9,19,20,21]. However, findings are not uniform—some OPSCC series report no significant baseline ADC difference by HPV—underscoring that protocol choices (b-value spread, readout/fat-suppression, and segmentation) can mask or magnify biological effects [8]. In HPV-positive OPSCC, ΔADC at week 2/week 4 is the most actionable early biomarker for outcome-oriented decisions (e.g., surveillance intensity and adaptive planning), while baseline ADC/ADC-radiomics are best used as a phenotypic context that can refine risk estimates when methods are explicitly standardised [8,9,11,18,19,20,21,22,23].

Baseline ADC is available upfront and is, therefore, attractive for initial stratification (e.g., supporting risk phenotyping or de-escalation eligibility), but it is also more vulnerable to inter-protocol variability and can represent mixed processes (cellularity, oedema, and necrosis), contributing to inconsistent prognostic strength across cohorts and potential attenuation after adjustment for HPV/p16 status and other clinical covariates [8,10,20,22]. By contrast, midtreatment ΔADC (dADC) functions as an early response biomarker, capturing therapy-induced microstructural change before gross anatomical response, and is, therefore, more directly actionable for response-adaptive treatment modification (escalation or de-intensification) when measured at a predefined timepoint [11,18,22]. Practically, this supports a two-stage interpretation: baseline ADC is best positioned as a pretreatment adjunct that must prove its incremental value beyond clinicopathologic predictors within HPV-positive cohorts, whereas midtreatment dADC is best positioned as a trigger candidate for adaptive protocols—where the key evidentiary requirement is prospective validation of a pre-specified timepoint and threshold linked to an actionable decision rule and robust cross-centre standardisation [14].

Key clinical DWI/ADC evidence is summarised in Table 1, including prospective serial-treatment cohorts defining early response timepoints and contemporary HPV-focused analyses that clarify the comparative utility of baseline ADC versus midtreatment ΔADC for clinical decision-making.

### 3.2. MRI Radiomics

#### 3.2.1. Reproducibility, Standardisation, and Model Credibility

MRI radiomics refers to the extraction of quantitative image biomarkers from routine MRI to characterise tumour phenotypes in a reproducible way [14]. Typical pipelines comprise standardised image acquisition (e.g., CE-T1, T2, DWI) with key parameters reported; tumour/ROI segmentation (2D or 3D; manual or semi-automatic) with software and editing rules documented; preprocessing to place images on a comparable intensity scale and voxel size (normalisation, resampling, and optional registration/denoising); feature extraction using IBSI-compliant definitions for first-order (intensity histogram), shape, and texture families (GLCM, GLRLM, GLSZM, NGTDM), often with wavelet or Laplacian-of-Gaussian filter expansions; and finally, feature selection, model building (e.g., regularised regression or tree-based methods), and performance evaluation with prespecified cross-validation and, where possible, external validation in line with radiomics quality recommendations [14,15]. In HPV-associated OPSCC, the MRI radiomics studies referenced in this section broadly follow this framework: features are derived mainly from CE-T1, T2, and DWI/ADC within primary tumour (±nodal) ROIs, with pipelines that include intensity normalisation, voxel resampling, stability and redundancy filtering, explicit handling of class imbalance, and cross-validated model fitting [4,13,27,28]. Within such standardised workflows, MRI radiomics yields descriptors that plausibly reflect cellularity, stromal reaction, necrosis, and microarchitecture and can support HPV status classification and prognostic modelling when methods are transparently reported and quality criteria are met [4,13,15,27,28]. 

Across modalities, a recurring barrier to clinical translation is **reproducibility**. Feature values vary with acquisition, reconstruction, segmentation policy, and preprocessing; therefore, adherence to the **Image Biomarker Standardisation Initiative (IBSI)** for feature definitions, explicit reporting of diffusion b-values and ΔADC calculation, and use of harmonisation methods (e.g., ComBat) are essential [14]. Guidance from **EIBALL/EORTC** outlines validation tiers, endpoints, and reporting for biomarker studies, and Quantitative Imaging Biomarkers Alliance **(QIBA) diffusion profiles** support scanner performance for DWI [29,30]. Radiomics quality assessments continue to find modest Radiomics Quality Scores and limited external validation, underscoring the need for **transparent pipelines, pre-registration, and independent testing** [15].

#### 3.2.2. HPV Phenotype Classification from MRI

Diffusion-derived radiomic features repeatedly emerge as informative for HPV phenotype discrimination. In a focused DWI study of pretreatment OPSCC with p16-defined HPV status, Lenoir et al. acquired 3T DWI with six b-values (0, 50, 100, 500, 750, 1000 s/mm^2^); whole-tumour ADC histograms showed that kurtosis on ADC_{b0–1000} separated HPV-positive from HPV-negative disease with AUC ≈ 0.89, and the authors emphasised that separability depended on the b-value scheme used to compute ADC [20]. Building on this diffusion signal in a full radiomics framework, a single-centre multiparametric MRI study reported mean test AUC ~ 0.77 for HPV classification when ADC-derived features were included alongside CE-T1 and T2, indicating that diffusion features add measurable discriminative power beyond conventional sequences [13]. Extending to heterogeneous, multi-centre data with explicit class-imbalance handling, an ADC-only radiomics model performed at least as well as—and in some external-testing analyses better than—CE-T1/T2-based models, supporting the generalisability of diffusion-anchored HPV phenotyping when pipelines are transparently validated [28]. Taken together, these results suggest that, within MRI radiomics workflows, much of the separative signal for HPV status resides in diffusion, provided ADC is computed with appropriate b-values and models are developed and tested with attention to class balance and external validation [13,20,28].

#### 3.2.3. Prognostic Models and De-Intensification

Beyond phenotype classification, MRI radiomics is also predictive of clinical outcome. In a large single-centre OPSCC cohort, Boot et al. manually delineated 249 primary tumours on pretreatment native T1-weighted MRI and extracted 498 radiomic features for HPV status classification and overall survival modelling [27]. Radiomics-only logistic regression and random forest models achieved an AUC of 0.79 for HPV prediction, whereas a model combining radiomic factors with clinical parameters improved HPV classification to an AUC of 0.89 and yielded an overall survival C-index of 0.72 [27]. This supports MRI radiomics as an augment to, rather than a replacement for, established clinicopathologic risk factors in OPSCC.

In HPV-positive neoadjuvant-chemotherapy cohorts, Lyu et al. conducted a retrospective multicohort study of p16-positive OPSCC in which primary-plus-nodal MRI radiomics were used to predict neoadjuvant chemotherapy response and to stratify survival in an independent validation cohort, supporting response-oriented risk stratification in HPV-positive disease [4]. Together with the large single-centre series by Boot et al. [27], these findings support MRI radiomics as an augmentation to clinicopathologic factors rather than a replacement. However, historical imaging observations require cautious reinterpretation because of confounding caused by subsite mix, protocol heterogeneity, and incomplete HPV stratification.

Between-scanner and protocol differences can shift radiomics feature values—not just the visual appearance of images. In MRI radiomics, this is critical because classifiers rely on subtle intensity and texture patterns; if those patterns mainly capture scanner or protocol differences rather than tumour biology, models may appear to perform well in single-centre experiments but fail when applied to new sites. First-order intensity features (e.g., mean, median, and entropy) and texture features (e.g., GLCM contrast/entropy, GLRLM run-lengths, GLSZM zone sizes, and NGTDM coarseness) are particularly sensitive to scanner/vendor and field strength, coil configuration, sequence/readout, fat suppression, intensity normalisation and binning, and voxel size/resampling; ADC-derived features additionally vary with the b-value scheme and fitting model [14]. To limit these effects, the IBSI recommends standardised feature definitions and full reporting of acquisition and preprocessing (scanner/vendor, key sequence parameters, all b-values, segmentation policy, and normalisation/resampling) [14]. For multi-centre HPV-associated OPSCC radiomics, statistical harmonisation methods such as ComBat can further mitigate scanner- and site-related shifts in feature distributions and improve comparability across centres, as shown in multi-centre imaging and head and neck radiomics studies [31,32]. Wherever possible, test–retest or phantom data should be used to identify which features remain stable in the chosen pipeline, ensuring that downstream HPV classification or prognostic models are driven by robust biological signals rather than hidden protocol differences [14,31,32].

For outcomes, radiomics is most persuasive when combined with clinicopathologic factors rather than used in isolation. In a large single-centre OPSCC cohort, Boot et al. showed that T1-based MR radiomics predicted HPV status with AUC ~0.79 and that adding radiomic factors to clinical variables improved HPV-classification (AUC 0.89) and overall survival prediction (OS C-index ~ 0.72) [27]. In HPV-positive neoadjuvant chemotherapy cohorts, Lyu et al. used baseline MR radiomics to predict NAC responses across internal and external validation sets and then constructed nomograms that combined radiomic signatures with clinical characteristics to stratify PFS and OS [4]. Together with ADC-focused classification work showing that diffusion-derived features often carry the strongest HPV-separating signal [13,20,28], these studies support a pragmatic approach: we should leverage ADC-anchored radiomics to assist HPV phenotyping, and integrate radiomic signatures with established clinical factors to refine risk stratification, provided that acquisition, segmentation, and validation are standardised and transparently reported [4,27].

Representative MRI radiomics pipelines and validation approaches are summarised in Table 2, spanning HPV classification and prognostic modelling, with feature definition and reporting expectations aligned with IBSI guidance.

### 3.3. CT Radiomics and Deep Learning Models

#### 3.3.1. CT Radiomics for HPV Phenotype Classification

Early feasibility work showed that CT texture differs between HPV-positive and HPV-negative primaries [16], with Buch et al. demonstrating on CT that hand-crafted texture features from tumour ROIs separate groups when referenced against p16/HPV status—establishing a reproducible radiomic signal on routine scans [16]. This was followed by multi-centre development with external validation, where a CT radiomic signature for p16/HPV achieved AUCs around 0.70–0.80 across scanners and institutions [13,33]. Independent cohorts then corroborated HPV discrimination using CT radiomics in both primary tumours and metastatic nodes—linking heterogeneity metrics to HPV status in OPSCC [34,35]—while related methodological work examined pipeline generalisability/robustness [36]. Fully automated 3D models were introduced to reduce manual annotation and capture contextual cues on CT [37], and combined radiomics with HPV status to enable risk stratification beyond imaging alone [37]. Together, these studies support CT radiomics as a scalable tool for HPV phenotyping, with complementary information available from nodal analyses and evolving workflows that emphasise robustness and clinical utility [16,34,35,36,37,38].

#### 3.3.2. Prognostic CT Radiomics and Nodal/Peritumoural Features

CT radiomics—especially when combined with clinical variables—improves predictions of OS/PFS and locoregional control over clinical factors or stage alone [39]. In 2020 [39], pretreatment contrast-enhanced CT radiomics was developed and validated to classify HPV status and stratify overall survival; when radiomics was fused with clinical variables, survival prediction outperformed clinical-only or stage-only models, supporting integration of imaging-derived phenotypes into risk prediction [39].

Larger OPSCC series relate pretreatment CT texture to recurrence and control, and adding peritumoural rings to intratumoural features improves prediction and yields disease-free survival (DFS) nomograms that are straightforward to apply clinically [40,41]. In [40], primary tumour textures on planning CT were associated with local recurrence, indicating an independent failure-risk signal from radiomic heterogeneity [40]. In [41], CT features associated with HPV status also informed prognosis, and combining intratumoural with peritumoural features improved discrimination and supported clinically usable DFS nomograms [41].

Methodological decisions materially shape radiomic performance. In Ren and Yuan’s CT radiomics study, 3D segmentations produced a higher proportion of reproducible texture features than 2D, yet HPV classification accuracy was actually slightly better with 2D models—illustrating that more complex volumetric segmentation does not necessarily improve prediction and that practicality and endpoint performance must be considered alongside feature stability [42]. Consistent with this, radiomics quality audits and OPSCC-specific texture reviews highlight that heterogeneous segmentation strategies and incomplete reporting of ROI definitions (for example, gross tumour versus post-contrast enhancing volume), reconstruction kernels, and post-processing parameters make it difficult to reproduce and externally validate models across centres [24,43].

Kann et al. retrospectively evaluated a previously developed CT-based deep learning model within the ECOG-ACRIN E3311 randomised de-escalation trial cohort. Pretreatment contrast-enhanced CTs were used; on each scan, the largest pathologic node (by short axis) and up to two additional nodes were segmented and labelled for extranodal extension (ENE) using surgical pathology as the ground truth. The algorithm produced node-level ENE probabilities and was benchmarked against four board-certified head and neck radiologists (blinded), with AUC as the primary endpoint. [3]. In 178 scans (313 nodes; 71 with ENE), the model achieved AUC = 0.86 (95% confidence interval (CI) 0.82–0.90), outperforming all readers (*p* < 0.0001). At the reader-matched specificity (false-positive rate ≈ 22%), algorithm sensitivity reached 75% (≈+13% absolute vs. the best reader); at a 30% false-positive rate, sensitivity was ≈90%. Reader specificity and sensitivity varied widely (43–86% and 45–96%, respectively) with poor inter-reader agreement (κ ≈ 0.32), underscoring the potential clinical utility of a consistent AI screener [3]. Because overt ENE was excluded by protocol in E3311, the cohort represents a diagnostically challenging, pre-operative screening population; the authors argue that this trial-anchored evaluation offers rare, high-credibility evidence for head and neck AI. Noted caveats include retrospective design and the need for node selection/segmentation; nevertheless, performance gains were most pronounced for >1 mm ENE and in nodes ≥ 1 cm short axis, aligning with clinically meaningful thresholds [3].

In interpretable pipelines, useful performance can be retained while keeping the decision process transparent. Altinok et al. used the Mens eX Machina selector to reduce 851 CT radiomic features to two shape descriptors: sphericity and max2DDiameterRow [44]. A Bayesian Network trained on these two features achieved AUC 0.78 in training and 0.72 on the held-out test set. The low-dimensional, conditional-probability structure made feature contributions explicit. A higher-capacity SVM using 25 features increased test AUC to 0.83 but reduced interpretability. Fanizzi et al. trained an Inception-V3 CNN on GTV-cropped CT images from the OPC-Radiomics cohort and evaluated it on an independent multi-centre test set [45]. They applied Grad-CAM to visualise salient regions. In correctly classified HPV-positive cases, saliency maps were predominantly intratumoural, whereas HPV-negative predictions emphasised tumour edges. The independent-test AUC was 0.735, showing that post hoc explainability can be combined with competitive predictive performance.

Methodological quality remains a constraint. In a focused systematic review of OPSCC-HPV radiomics, Spadarella et al. [15] reported a median Radiomics Quality Score of ~33% (range: 0–42%), with few studies performing external validation and no studies providing a public protocol, phantom study, or repeated-timepoint imaging. These findings underscore the need for transparent, standardised pipelines; preregistered analysis plans; and independent, multi-centre testing before clinical adoption. Accordingly, rigorous reporting and harmonisation are essential; the following checklist summarises the minimum items required for reproducible studies.

CT radiomics robustness is sensitive to acquisition and reconstruction choices, including reconstruction kernels, slice thickness, and denoising. This was highlighted in methodological guidance and reviews of OPSCC-HPV radiomics, as noted in both the IBSI consensus and the Spadarella et al. review, emphasising protocol standardisation and careful reporting to limit feature instability and bias [14,15].

To improve multi-centre portability, adherence to the IBSI reference feature definitions and processing scheme is recommended; the IBSI provides consensus definitions and reference values for a broad set of features and outlines validation practices that enhance reproducibility. In parallel, harmonisation strategies such as ComBat can reduce inter-centre shifts in feature distributions, supporting pooled modelling while maintaining biological signal [14].

For HPV-associated OPSCC, CT radiomics is most mature for HPV status classification and for augmenting prognostic models when fused with clinical variables; peritumoural features add value [16,24,33,34,35,37,38,39,40,41,42,46]. Deep learning extends this to segmentation-free status prediction and ENE triage on trial data [3,47]. Robust deployment depends on IBSI-consistent pipelines, harmonisation, and external validation [14,15].

CT radiomics and deep learning studies relevant to HPV phenotyping and nodal risk features are summarised in Table 3, from early CT texture signals through multi-centre HPV classifiers and recent trial-linked deep learning for clinically important endpoints such as ENE, alongside standardisation/validation frameworks intended to improve portability.

### 3.4. FDG-PET/CT and Texture Analysis

At diagnosis, FDG-PET/CT complements contrast-enhanced CT/MRI by improving whole-body staging (occult nodal and distant metastases), helping localise unknown primaries, and informing treatment intent. In radiotherapy workflows, it assists GTV delineation and highlights metabolically active nodes that may be equivocal on anatomical imaging alone. After chemoradiotherapy (CRT), a timed posttreatment scan (typically at 10–12 weeks) is used for response assessment: a complete metabolic response has a high negative predictive value and can obviate planned neck dissection, whereas residual or equivocal uptake prompts short-interval imaging, biopsy, or salvage. Outside this window, PET/CT is used selectively in surveillance when there is clinical or biochemical suspicion of recurrence. In HPV-associated OPSCC—where disease often presents with small primaries and nodal metastases, outcomes are favourable but heterogeneous, and HPV/p16 status and T/N stage do not fully capture risk—FDG-PET/CT contributes baseline metabolic metrics such as SUV_{max}/SUV_{mean}, metabolic tumour volume (MTV), total lesion glycolysis (TLG), and intratumour heterogeneity descriptors that reflect tumour biology beyond size. These parameters can refine risk stratification, aid early and post-CRT response assessment, and support posttreatment decisions (e.g., observation, biopsy, or salvage) while complementing MRI-based diffusion and texture information.

#### 3.4.1. Volumetric PET Metrics (MTV/TLG) and Prognosis

Across treatment-uniform CRT cohorts and larger single-centre series, volumetric PET metrics stratify risk. In a uniform intensity-modulated radiotherapy (IMRT) + concurrent-chemo cohort of 176 OPSCC patients, Lim et al. delineated MTV with a 42% SUVmax threshold and computed TLG as SUVmean × MTV; each doubling of primary tumour MTV predicted locoregional failure (HR ≈ 2.4; *p* = 0.005), distant metastasis, and overall survival (HR ≈ 1.9 and 1.8; *p* < 0.001). TLG was likewise associated with DM and OS (*p* < 0.001). In Kim et al. (n = 221), pretreatment MTV and TLG (primary tumour volume of interest (VOI)) were significant on univariate analysis, with empiric cut-offs (e.g., MTV ≈ 11 mL; TLG ≈ 79 g) separating DFS/OS; overall 5-year OS of 72% and DFS of 79.5% were reported, supporting baseline volumetric burden as a pragmatic class to prioritise for LRC, DM, PFS/DFS, and OS [49,50].

By contrast, in Lim et al., primary tumour SUVmax was associated with death on univariate analysis but lost significance after adjustment for T stage, whereas MTV and TLG remained independently associated with overall survival (*p* ≈ 0.013 for TLG and *p* ≈ 0.032 for MTV; SUVmax *p* ≈ 0.16) [49]. Kim et al. likewise found that age, tumour SUVmax, and MTV were independent predictors of OS and DFS in multivariable models [50]. Conceptually, SUVmax samples only the hottest voxel, whereas MTV and TLG encode whole-tumour metabolic burden, which likely explains why volumetric PET metrics often demonstrate stronger or more consistent prognostic associations than SUVmax across series. Emerging HPV-positive OPSCC cohorts suggest that volumetric and, in some studies, heterogeneity measures can further refine risk stratification, although findings remain heterogeneous and dependent on model specification.

#### 3.4.2. Intratumoural Heterogeneity and Texture-Derived Risk

Within HPV-positive OPSCC cohorts, volumetric FDG burden generally remains prognostic, but cut-offs and effect sizes are often more modest and variable than in mixed HNSCC series. Alluri et al. studied 70 patients with HPV-positive stage III–IV OPSCC and found that total and primary tumour MTV were significantly associated with event-free survival, with total MTV and then primary MTV remaining independent prognostic markers in multivariable models, whereas SUV metrics and most TLG measures did not [51]. In Mena et al. (n = 105 HPV-positive OPSCC), baseline MTV measured on PET/CT using gradient-based and 50% SUV_{max} segmentation showed an optimal total MTV cut-off of ≈12.7 mL for event-free survival in Kaplan–Meier analysis, while multivariable models identified intratumoural heterogeneity (AUC-CSH) as an independent predictor; patients with both higher heterogeneity and higher SUV_{max} had the worst outcomes [52]. Together, these data suggest that in HPV-positive OPSCC, tumour burden remains clinically informative, but prognostic thresholds and independent effects are more nuanced and depend on how volumetric and heterogeneity metrics are modelled.

Methodological details are useful for replication, as shown when Alluri et al. focused on stage III–IV HPV-POSITIVE OPSCC treated definitively, analysing PET-derived SUVmax/SUVmean/SUVpeak, MTV, TLG, and reporting MTV as independent prognosticators after adjustment [51]. Mena et al. computed primary tumour MTV via gradient-based and 50% SUVmax segmentation and defined TLG as SUVmean × MTV; optimal cut-offs (e.g., SUVmax ~ 16.7; MTV ~ 12.7 mL) separated risk groups, and heterogeneity (lower AUC-CSH) plus higher MTV identified the poorest EFS, underscoring that volumetric burden remains valuable even as HPV-positive biology moderates effect sizes [52].

#### 3.4.3. PET Texture and Radiomics-Based Prognostic Models

In advanced T-stage OPSCC treated with curative-intent (chemo)RT, pretreatment PET texture adds prognostic signal beyond bulk uptake. Cheng et al. extracted histogram, GLCM (e.g., uniformity, entropy, and dissimilarity), and NGTDM features from primary tumour PET VOIs (SUV ≥ 2.5) [53]. On multivariable Cox analysis, GLCM uniformity remained independently associated with PFS/DSS/OS alongside TLG; a simple score combining TLG > 121.9 and uniformity ≤ 0.138 identified markedly poorer outcomes (*p* < 0.001 for PFS/DSS; *p* = 0.002 for OS). These findings illustrate that spatial texture—particularly low uniformity (i.e., higher heterogeneity)—can stratify risk independent of volumetric burden.

Within HPV-positive OPSCC, heterogeneity metrics retain prognostic value and refine volumetric risk. In 105 HPV-positive OPSCC patients, Mena et al. quantified intratumoural heterogeneity using the AUC-CSH index (where lower AUC-CSH indicates higher heterogeneity) and showed that AUC-CSH was an independent predictor of event-free survival, while Kaplan–Meier analysis identified optimal baseline cut-offs of SUV_{max} ≈ 16.7 and total MTV ≈ 12.7 mL that separated risk groups [52]. Patients with both higher heterogeneity and higher MTV, or higher heterogeneity and higher SUV_{max}, had the worst EFS, supporting the view that texture-derived heterogeneity captures biologically meaningful spatial variation not fully reflected by MTV/TLG alone in HPV-positive disease [52].

In a multi-institutional OPSCC cohort (n = 311, Yale + TCIA), Haider et al. extracted 1037 PET and 1037 CT radiomic features from primary tumours and nodes and built random survival forest models using three inputs: AJCC-8-only, radiomics-only, and combined (AJCC + radiomics) [2]. In HPV-positive disease, the best radiomics model achieved a C-index of ≈ 0.62 for PFS versus ≈ 0.54 for AJCC alone, with radiomics and combined models generally outperforming AJCC in time-dependent analyses for PFS (and often OS), indicating that clinical + radiomics models provide an added prognostic value beyond staging and standard covariates. To assess generalisability and guard against single-centre optimism, we next consider multi-centre benchmarking and blind-test challenges.

Where available, multi-centre blind-test benchmarks such as HECKTOR are particularly informative. These challenges aggregate PET/CT data from several institutions, apply a common preprocessing and labelling scheme, and keep test labels hidden so models are evaluated on a truly unseen, multi-centre cohort [54]. Demonstrating good performance under these conditions shows that radiomics and AI models can generalise beyond their development site, helping to mitigate single-centre overfitting and providing a realistic benchmark for clinical translation.

#### 3.4.4. Multi-Centre Benchmarks

While benchmarks demonstrate technical feasibility, translation ultimately depends on practice-changing clinical evidence, exemplified by PET-NECK. In a multi-centre randomised trial, PET-CT–guided surveillance after CRT was non-inferior to planned neck dissection for overall survival, dramatically reducing operations (54 vs. 221 neck dissections) and yielding cost savings of approximately GBP 1492 per patient, with similar complication rates and quality of life—a practice-changing result in a predominantly oropharyngeal, p16-positive (HPV-positive) cohort [5].

In parallel, shared multi-centre datasets and challenge platforms help define current performance ceilings and reinforce the case for rigorous standardisation. The TCIA head and neck FDG-PET/CT collections provide openly available multi-centre data, while the HECKTOR challenges offer multi-centre, blind-test benchmarks in which fully automated PET/CT pipelines tackle tumour segmentation and progression-free survival prediction. In the 2021 edition, the best methods achieved Dice ≈ 0.76 for primary tumour segmentation and C-index ≈ 0.70–0.72 for PFS on a held-out multi-centre test set, illustrating that robust external performance is achievable but still leaves headroom for further refinement and standardisation [54].

PET radiomics are highly susceptible to scanner and reconstruction choices (e.g., reconstruction kernel, iterations, post-reconstruction filters, and voxel size), and numerous head and neck PET radiomics papers have emphasised that inconsistent acquisition and preprocessing can markedly alter feature values and model performance. Studies from Payabvash and others on head and neck cohorts illustrate that voxel-intensity normalisation and consistent preprocessing pipelines can materially change which radiomic features are selected as important and can improve prognostic model performance compared with using raw SUV values alone [55]. Conceptually, pre-specifying the normalisation method and keeping reconstruction parameters fixed helps to reduce spurious variability and should yield more reproducible, generalisable signatures, particularly in multi-centre OPSCC radiomics.

Differences between fixed and relative SUV thresholds can alter radiomic values and downstream risk groups. Cheng et al. explicitly thresholded primary tumour VOIs using SUV ≥ 2.5 and still found GLCM-derived uniformity to be independently prognostic alongside TLG, illustrating that while segmentation choices modulate feature values, they do not abolish prognostic texture signal [53]. Multi-centre blind-test challenges such as HECKTOR further show that fully automatic PET/CT segmentation and outcome-prediction models achieve only moderate performance and struggle in more difficult cases, reinforcing that standardised preprocessing, consistent VOI definitions, and rigorous external validation are essential, along with feature stability testing and parsimonious, interpretable models [54].

Clinically credible PET models in HPV-associated OPSCC, therefore, combine clinical covariates with volumetric PET metrics (and, where robust, texture features), are trained on standardised or harmonised data, and demonstrate performance on independent test sets. Single- and multi-centre OPSCC series show that radiomics can add prognostic value beyond AJCC-8 [2,49,50,52,53], while multi-centre blind-test platforms such as HECKTOR and randomised data such as PET-NECK illustrate the importance of external validation and reproducibility for clinical adoption [5,54,55].

Key FDG-PET/CT metrics, radiomics models, and response frameworks are summarised in Table 4, including baseline metabolic burden studies, PET-guided management evidence, response standardisation, and multi-centre benchmarking efforts.

### 3.5. Methodological Issues Driving Heterogeneity

Throughout this review, we have referenced and discussed methodological issues that are driving heterogeneity in reported performance. In Table 5, we summarise these issues to create a framework for future research in this field. Reproducible quantitative imaging in HPV-positive OPSCC depends on transparent, standardised reporting across acquisition, segmentation, feature extraction, and validation because small methodological differences (e.g., diffusion b-value selection, ROI rules, or inter-site harmonisation) can materially alter reported performance. We, therefore, contextualise current radiomics quality limitations and reporting gaps [14,15,24] and align the checklist with practical approaches to reducing between-site variability [31,32] and modality-specific protocol sensitivity (e.g., b-value dependence in diffusion biomarkers) [20]. For PET-derived endpoints and response assessment, we additionally reference standard response frameworks to support consistent reporting in longitudinal workflows [17,57].

## 4. Future Directions

As discussed in this article, quantitative imaging in HPV-positive OPSCC is an area of active research and development globally, and several future directions are particularly promising. Serial intratreatment DWI on MR-Linac platforms consistently shows mid-course ADC rises in responding head and neck disease [11,18,22], supporting adaptive designs that use pre-specified ΔADC thresholds to modify target volumes and doses within a single course of CRT. Recent prospective MR-Linac work demonstrates the feasibility of weekly DWI acquisition and quantifies temporal ADC change at whole-tumour and subvolume levels, providing an operational footing for response-adapted radiotherapy in HPV-positive OPSCC [22]. In parallel, PET-CT-guided post-CRT surveillance has already proven non-inferior to planned neck dissection in PET-NECK [5] while markedly reducing operations, and next-generation studies should integrate baseline PET burden (e.g., MTV/TLG) [49,50,52] and PET radiomics [2,55] with PET-guided surveillance schedules to personalise follow-up intensity in HPV-positive populations, formally testing health–economic and quality-of-life endpoints alongside oncologic outcomes. For radiomics to influence guidelines, pipelines must also be prospectively specified and auditable, aligned with trial-readiness criteria such as those articulated by EIBALL/EORTC (EIBALL/EORTC imaging biomarker guidelines)—including pre-registration, clearly defined endpoints, data-management plans, and decision-impact analyses—so that imaging biomarkers can be embedded credibly into de-intensification and adaptive-RT trials.

Moving from proof-of-concept performance to clinical adoption in HPV-positive OPSCC now requires a coordinated translational programme that prioritises generalisability, decision impact, and workflow readiness. In the short term (12–24 months), the field should converge on a small number of “trial-ready” biomarkers per modality and agree upon base definitions (acquisition parameters, preprocessing, and ROI rules); mandate site-separated external validation as a minimum evidentiary threshold; and adopt transparent, pre-registered pipelines aligned with reproducibility standards and radiomics quality recommendations [14,15]. Given known scanner/protocol effects, harmonisation and technical QA (including ComBat-style approaches and diffusion performance profiling) should be used where appropriate to support multi-centre pooling and comparability [14,31,32]. In the medium term (2–5 years), candidate biomarkers should be embedded prospectively as decision triggers within multi-centre de-escalation and adaptive radiotherapy designs—supported by the feasibility of serial intratreatment DWI on MR-Linac platforms and repeated evidence that midtreatment ΔADC captures clinically meaningful response during treatment. In parallel, surveillance pathways provide a pragmatic translation target: PET-CT-guided follow-up has already reduced unnecessary operations without compromising outcomes in PET-NECK, and future studies should test whether baseline PET burden (MTV/TLG) and PET radiomics can personalise surveillance intensity with health–economic and quality-of-life endpoints [2,5,54]. In the long term (5+ years)**,** translation will require auditable, trial-ready workflows (pre-specified endpoints, data governance, and decision-impact evaluation) aligned with frameworks such as EIBALL/EORTC, enabling biomarkers to be embedded credibly into adaptive-RT and de-intensification trials.

## 5. Conclusions

Quantitative imaging in HPV-positive OPSCC has moved beyond exploratory correlations to a small number of convergent, clinically meaningful signals. Across prospective head and neck cohorts, midtreatment ΔADC rises in DWI are repeatedly associated with better local control and survival and outperform baseline ADC once HPV status and stage are accounted for [11,18,22]. PET studies show that volumetric metrics such as MTV and TLG are more robust prognosticators than SUVmax alone, linking higher metabolic burden to worse locoregional control, distant metastasis, and overall survival in both mixed HNSCC and HPV-positive OPSCC [48,49,51,52]. Radiomics and AI models—based on MRI, CT, and PET—consistently add incremental value when combined with clinicopathologic factors, improving HPV status discrimination and progression-free or overall survival prediction beyond AJCC-8 staging in multi-institutional series [2,3,4,16,27]. At the same time, systematic reviews highlight low median Radiomics Quality Scores, limited external validation, and heterogeneous methodology, underscoring that these promising tools are not yet ready for guideline-directed use [15].

## Figures and Tables

**Table 1:** Diffusion-weighted MRI (DWI) and apparent diffusion coefficient (ADC) in HPV-associated OPSCC.

Author	Year	Study Type	Objectives	Analysis	Results (AUC, b Values, etc.)	Cohort	Main Outcomes
Vandecaveye et al. [11]	2010	Prospective observational	On-treatment ΔADC during CRT as early response biomarker	Not reported; whole-tumour/ROI ADC metrics; univariate and ROC analyses	Serial ΔADC during CRT (weeks 2 and 4) significantly higher in complete responders; early ΔADC correlated with 2-year local control (univariate).	n = 37 baseline; n = 30 with on-treatment DWI; HNSCC, including OPSCC	On-treatment increases in ADC at weeks 2–4 tracked response. Established feasibility and timing of early DWI as a response biomarker in HNSCC.
Vandecaveye et al. [18]	2012	Prospective observational	Early post-CRT DWI to monitor treatment response	Not reported; threshold-based ADCmean and ROI analysis; diagnostic accuracy (ROC)	Early post-CRT DWI: Primary-site PPV ≈ 80% and NPV ≈ 100% for residual tumour; Nodal PPV ≈ 36% and NPV ≈ 91%. Six b-values up to b = 1000 s/mm^2^.	n = 29 HNSCC with DWI 2–4 weeks after CRT	Early posttreatment ADC identified residual primary disease with very high NPV. Supports DWI as a safe triage test to avoid unnecessary early salvage surgery/biopsies.
King et al. [12]	2013	Observational	Baseline/early-treatment ADC predicting CRT response	Not reported; ROI/histogram ADC; group comparisons; logistic modelling	Pretreatment ADC and early ΔADC (week 3) differentiated complete vs. incomplete responders (*p* ≈ 0.004 for ΔADC); superior to size change early in CRT.	n = 56 baseline; ~n = 37 had serial DWI during CRT; HNSCC mixed sites, including OPSCC	Early ΔADC predicted CRT response better than anatomic size. Supports integrating interim DWI into adaptive pathways.
Schouten et al. [8]	2015	Retrospective observational	Baseline ADC vs. HPV status	Not reported; whole-tumour VOI; *t*-tests/ANCOVA/regression	HPV-positive tumours had lower mean ADC than HPV− (1.00 vs. 1.18 × 10^−3^ mm^2^/s; *p* = 0.003). No AUC reported.	n = 44 OPSCC (28 HPV-POSITIVE/16 HPV−)	Baseline ADC differs by HPV status. Cautions against using absolute ADC as a universal prognostic marker without HPV stratification.
Zhang et al. [25]	2015	Retrospective observational (NPC, non-OPSCC)	ADC prognostic value in nasopharyngeal carcinoma	Not reported; ROI ADC; ROC and survival analyses	ADC metrics predicted outcome in nasopharyngeal carcinoma.	NPC cohort (non-OPSCC)	ADC associated with outcomes in NPC. Cross-site diffusion precedent in SCC but non-OPSCC—use as supportive context only.
Lenoir et al. [20]	2022	Cross-sectional	HPV/p16 classification with ADC histogram metrics	Not reported; ADC maps and histogram/heterogeneity metrics; ROC/AUC	ADC histogram kurtosis on b0–1000 distinguished HPV-POSITIVE vs. HPV− with AUC = 0.893, sensitivity 100%, specificity 82.6%; b-value choice critically affected separability.	n = 34 OPSCC (23 HPV-POSITIVE/11 HPV−)	Kurtosis using b0–1000 provides strong HPV discrimination. Demonstrates acquisition/ADC calculation choices (include b = 0) change biomarker performance.
Ravanelli et al. [9]	2018	Observational	Pretreatment ADC distributions vs. HPV and outcomes	Not reported; ADC + texture features; logistic modelling	Pretreatment mean ADC lower in HPV-POSITIVE than HPV− (median ~0.85 vs. 1.03 × 10^−3^ mm^2^/s). Combined model (ADC + clinical) achieved sensitivity ~83% and specificity ~93% for HPV status.	n ≈ 59 OPSCC with known HPV	ADC and selected textures correlate with HPV status and outcome. Supports multiparametric models over single metrics.
Ravanelli et al. [19]	2020	Retrospective observational (single-centre)	Pretreatment ADC histogram predicting outcome (HPV-aware)	Not reported; histogram analysis; Cox proportional hazards with FDR correction	Pretreatment ADC histogram metrics associated with PFS/OS; in HPV− subgroup, entropy predicted OS (HR ≈ 4.85, *p* = 0.01).	n = 59 advanced OPSCC; 28 HPV-POSITIVE/31 HPV−; ≥2-year follow-up	ADC heterogeneity carries prognostic information, particularly in HPV− disease. Heterogeneity metrics may complement HPV stratification.
Freihat et al. [21]	2021	Observational (PET/MRI)	Cross-modal PET/DWI parameters for HPV and response	Not reported; ROI ADC; ROC/AUC; PET/MRI coregistration	Pretreatment ADCmean lower in HPV-POSITIVE vs. HPV− (cut-off ~0.80 × 10^−3^ mm^2^/s; sensitivity ~77%, specificity ~72%). FDG PET metrics (SUVmax/MTV/TLG) not associated with HPV status.	n ≈ 40 primary OPSCC	ADC outperformed FDG metrics for HPV discrimination. Supports DWI as the MRI component most informative for HPV phenotyping.
Bollen et al. [22]	2024	Prospective observational	HPV-aware ΔADC midtreatment; HPV status modelling	Radiomic extraction of 105 DW-MRI features; multivariable modelling with ROC/AUC	Week-4 ΔADC higher in HPV-POSITIVE vs. HPV− (≈95% vs. 55%, *p* < 0.01). Multivariable model (smoking, alcohol, GLCM-correlation, ADCmean, and 10th-percentile) predicted HPV with AUC = 0.77 (95% CI 0.70–0.84).	n = 178 prospectively collected OPC with pre-RT and week-4 DWI	HPV-POSITIVE tumours show larger early ΔADC; ΔADC predicted locoregional control. Reinforces ΔADC as a biologically plausible early-response and HPV-linked marker in large cohorts.
Boeke et al. [23]	2025	Prospective feasibility (MR-Linac)	Serial weekly DWI during RT	Not reported; longitudinal non-parametric tests; repeatability coefficients	MR-Linac weekly DWI: median ADC increased 49% (primary) and 24% (nodes) by end-RT; ADC changes exceeded repeatability thresholds by weeks 2–4.	n = 27 prospectively imaged on 1.5T MR-Linac; weekly DWI	Online DWI on MR-Linac is feasible and shows early ADC rise. Enables trial designs for ADC-guided online adaptation (timing around week 3).
Chan, Higgins, and Enepekides et al. [26]	2017	Observational (single-centre)	Baseline ADC differences based on HPV in primaries and nodes	Not reported; multivariable logistic regression; ROC analyses	Primary ADC + nodal ADC predicted HPV status: AUC ≈ 0.85 (primary), 0.90 (nodes); combined model AUC ≈ 0.92.	n = 40 OPSCC (28 HPV-POSITIVE/12 HPV−)	Combining nodal and primary ADC improves HPV classification. Metastatic-node ADC adds complementary signal to primary tumour metrics.
Sistonen et al. [10]	2021	Retrospective observational	ADC prognostic value with HPV adjustment	SPSS v25; Cox models; inter-observer agreement statistics	Higher pretreatment ADC associated with poorer DFS on univariable analysis; association disappeared after adjusting for p16. Breaks in RT and larger nodal volume independently predicted worse DFS.	n = 67 OPSCC (55 p16+); median follow-up 38 months	p16 confounds ADC-prognosis relationships in OPSCC. Stresses HPV-aware modelling and controlling for clinical covariates.
Zwanenburg et al. [14]	2020	Standards/methods (IBSI)	Standardisation of radiomics features	IBSI reference definitions; compliance checklists	Consensus definitions for feature calculation; reporting recommendations for b-values, motion control, and ROI policies in DWI radiomics.	Consensus methodology paper	Standardised feature definitions mitigate inter-site variability. Necessary for reproducible DWI radiomics and for multi-centre pooling.

**Table 2:** MRI Radiomics for HPV status and prognosis in OPSCC: overview of multiparametric MRI radiomics pipelines (CE-T1, T2, ADC), segmentation and feature extraction, modelling approaches, performance metrics (AUC/C-index), cohorts, and key findings for HPV/p16 classification and survival modelling, with attention to feature stability and validation. Content aggregates evidence beyond what is detailed in the article text to provide a broader landscape of relevant studies.

Author	Year	Study Type	Objectives	Software Used	Results (AUC, b Values, etc.)	Cohort	Main Outcomes
Suh et al. [13]	2020	Retrospective observational	Multiparametric MRI radiomics for HPV classification	Segmentation: MITK; registration: SPM12; feature extraction: MATLAB (custom radiomic features); machine learning: scikit-learn and XGBoost; cross-validation: repeated stratified 3-fold, 20 repetitions, with grid search.	Using all sequences: logistic regression mean AUC 0.77 ± 0.12; random forest 0.76 ± 0.12; XGBoost 0.71 ± 0.12. Average sensitivity 0.71 and specificity 0.72 for logistic regression.	n = 60 (48 HPV-POSITIVE, 12 HPV−)	Adding ADC-derived features improved multiparametric MRI HPV classification (test AUC ~ 0.77). Supports the centrality of diffusion in MRI radiomics and the value of multiparametric fusion.
Zwanenburg et al. [14]	2020	Standards/methods (IBSI)	Feature definition standardisation and benchmarking	Not applicable—Image Biomarker Standardisation Initiative (IBSI) reference for standardised radiomic feature definitions and benchmarking.			IBSI provides standardised feature definitions and cross-software benchmarking for radiomics. Underpins reproducibility and multi-centre comparability—essential for MRI radiomics to generalise.
Freihat et al. [21]	2021	Observational (PET/MRI)	Cross-modal association of PET and DWI parameters with HPV and response	Software not reported. PET/MRI integration analysing associations between diffusion parameters (ADC) and PET metrics with HPV status and treatment response.	ADCmean significantly lower in HPV-POSITIVE; cut-off ≈ 0.80 × 10^−3^ mm^2^/s yielded sensitivity 76.9% and specificity 72.2% for HPV-POSITIVE. PET metrics (SUVmax, TLG, MTV) were not associated with HPV status; TLG- and MTV-predicted response.	n = 33 (pre- and 6-month post-CRT PET/MRI)	Lower pretreatment ADC in HPV-positive patients was associated with better CRT response in a PET/MRI setting. Cross-modal consistency (metabolism + diffusion) strengthens biological plausibility for MRI radiomics signals.
Lenoir et al. [20]	2022	Cross-sectional	HPV/p16 classification with ADC histogram metrics	MATLAB used to compute ADC maps and histogram/heterogeneity metrics with b-values 0–1000 s/mm^2^; no machine learning classifier was reported (metric-based analysis).	Best discrimination on ADC b0–1000: kurtosis AUC 0.893; sensitivity 100%; specificity 82.6%. b-values evaluated: 0, 50, 100, 500, 750, 1000 s/mm^2^; perfusion-sensitive maps (incl. b0) outperformed perfusion-insensitive maps.	n = 34 (11 HPV-POSITIVE, 23 HPV−)	ADC kurtosis/skewness (b0–1000) discriminated HPV status with AUC ≈ 0.89. Diffusion histogram features (beyond mean ADC) capture HPV phenotype on routine MRI; requires explicit b-value reporting.
Boot et al. [27]	2023	Single-centre retrospective (large OPSCC)	Prognostic modelling and HPV classification using MRI radiomics + clinical covariates	Software not reported. MRI radiomics combined with clinical covariates for HPV classification and prognostic modelling; specific segmentation, feature extraction, and learning libraries were not detailed in the abstract.	Radiomics-only models (LR/RF) AUC 0.79 for HPV classification. Combined clinical + radiomic factors AUC 0.89 for HPV status; overall survival C-index 0.72 (combined model).	n = 249 (91 HPV-POSITIVE, 159 HPV−)	Radiomics combined with clinical variables improved HPV classification and overall survival modelling over either alone. Positions MRI radiomics as an augmentation to clinicopathologic factors in OPSCC risk stratification.
Lyu et al. [4]	2024	Retrospective multicohort (HPV-positive NAC)	Response and prognosis stratification using primary + nodal MRI radiomics in HPV-POSITIVE NAC cohorts	Software not reported. Primary-plus-nodal MRI radiomics to predict neoadjuvant chemotherapy response in HPV-positive OPSCC; prognostic modelling framework described without naming specific software packages.	Validation survival stratification reported.		Primary–nodal MRI radiomics stratified chemotherapy response and survival on validation in HPV-positive cohorts. Suggests MRI radiomics can inform neoadjuvant decision-making in HPV-positive OPSCC.
Sim et al. [28]	2024	Multi-centre retrospective (external test)	HPV/p16 classification with MRI radiomics under class-imbalance handling	Software not reported. The study compared three feature selection methods, five class-imbalance strategies (including SMOTE and ADASYN), and twelve machine learning algorithms; the best external validation performance was achieved using random oversampling with ridge regression on the ADC-based model.	External validation: ADC radiomics with random oversampling + ridge regression AUC 0.791 (95% CI 0.775–0.808). Comparators: CE-T1WI AUC 0.604; T2WI AUC 0.695; CE-T1WI + T2WI AUC 0.642 (external set).	n = 154 total (training 110, external validation 44; 86% HPV-POSITIVE)	After rigorous class-imbalance handling, an ADC-only model outperformed CE-T1/T2 models on external testing. Indicates diffusion carries much of the separative signal for HPV status; robust validation and imbalance handling are critical.

**Table 3:** CT Radiomics and deep learning in OPSCC compilation of handcrafted CT radiomics and deep learning studies: study design, computational analysis, external validation where available, metrics (AUC/C-index), cohorts, and clinical endpoints (HPV classification, extranodal extension screening, local control/survival). The table intentionally extends past the works discussed in the manuscript to incorporate additional qualified studies for completeness.

Author	Year	Type of Study	Objectives	Software/Computational Analysis	Results (AUC/C-Index/etc.)	Cohort (n, Setting)	Main Outcomes—What They Found and Why It Matters
Buch et al. [16]	2015	Retrospective observational (single-centre)	Assess whether contrast-enhanced CT texture can discriminate HPV status in OPSCC.	Hand-crafted texture features (first-/second-order; GLCM); multivariate analysis; software not explicitly specified in paper.	Significant differences in multiple texture features between HPV-positive and HPV−; AUC not reported.	n = 100 OPSCC (46 HPV-POSITIVE, 54 HPV−); diagnostic/planning CT; single centre.	HPV-positive primaries showed more homogeneous CT texture (e.g., lower entropy, higher uniformity) than HPV−. Early evidence that standard planning CT encodes HPV biology, motivating later multi-centre HPV classifiers.
Leijenaar et al. [33]	2018	Multi-centre retrospective; development + external validation	Develop/validate a CT radiomic signature to predict p16/HPV status across institutions.	Hand-crafted radiomics per standardised definitions; feature selection and logistic regression; multi-centre harmonisation.	HPV classification AUCs ~0.70–0.80 across training/validation cohorts; survival model C-index ~ 0.63 for LRC in external validation (reported).	n ≈ 778 pooled across centres; standard planning CT; external multi-centre validation.	A CT signature predicted p16/HPV and related to outcomes with consistent performance across scanners. Demonstrated generalisability of CT radiomics for HPV phenotyping and prognosis beyond a single site.
Lee et al. [34]	2019	Retrospective; discovery + independent validation	Test whether CT texture of primary tumours and metastatic nodes can discriminate HPV status.	Texture extraction and logistic modelling; features included tumour entropy (primary) and intensity SD (nodes).	Discovery AUCs: 0.85 (primary), 0.82 (nodes); validation AUCs: 0.75 (primary), 0.70 (nodes).	Discovery: n = 95 primary/n = 91 nodes; Validation: n = 36 primary/n = 36 nodes; clinical CT.	Both primary tumour and nodal CT texture discriminate HPV, with independent signal from nodes. Supports leveraging nodal radiomics to complement primary tumour features for HPV classification.
Mungai et al. [35]	2019	Retrospective observational	Evaluate association of CT texture heterogeneity with HPV status.	First- and higher-order radiomics; *t*-tests and logistic regression; software not specified.	Multiple higher-order features differed by HPV status (*p* < 0.05); AUC not reported.	n = 50 OPSCC (35 HPV-POSITIVE, 15 HPV−); contrast-enhanced CT; single centre.	Heterogeneity metrics on CT associate with HPV positivity. Independent replication that simple CT-derived heterogeneity reflects HPV biology.
Choi et al. [39]	2020	Retrospective; development + dual external validation	Develop a CT radiomics model to predict HPV status and test whether a radiomics score adds to clinical OS prediction.	Three-dimensional segmentation; random-forest feature selection for HPV; Cox regression for OS; validation on TCIA.	HPV AUCs: 0.865 (train), 0.747 (test), 0.834 (external). OS C-index improved from 0.702 (clinical) to 0.733 (+radiomics); external OS C-index 0.866 and 0.720.	Train n = 61, test n = 25, external TCIA n = 78; planning CT.	CT radiomics predicted HPV status robustly and improved overall survival modelling beyond clinical factors. Demonstrates additive prognostic value and external validity, supporting clinical utility.
Ren et al. [42]	2020	Retrospective methodological comparison	Compare 2D vs. 3D segmentation pipelines for CT radiomics HPV classification in OPSCC.	Machine learning HPV classifier; random forests; SMOTE oversampling; comparison of 2D and 3D ROI approaches.	Reported strong HPV discrimination for both 2D and 3D pipelines; 3D did not consistently outperform 2D (exact AUCs vary by setting).	Single-institution OPSCC cohort; retrospective contrast-enhanced CT (exact n per paper).	Three-dimensional segmentation improved feature reproducibility but offered limited accuracy gains for HPV classification. Highlights practical pipeline trade-offs; 2D may be acceptable for classification with lower annotation burden.
Song et al. [41]	2021	Large retrospective; internal + external validation	Assess intratumoural and peritumoural CT radiomics for HPV classification and build a DFS nomogram for HPV-POSITIVE patients.	In-house radiomics (Haralick, Laws, CoLlAGe); MRMR feature selection; Cox modelling; clinicopathologic nomogram.	Accuracy for HPV prediction ~ 76% on validation; For HPV-positive DFS: RRS HR = 1.97 (95% CI 1.35–2.88); stage-specific HRs significant; nomogram C-index 0.59 (95% CI 0.54–0.65).	TCIA training n = 180/validation n = 282; external HPV-POSITIVE cohort (Cleveland Clinic) n = 120.	Peritumoural texture adds prognostic signal; a combined clinicopathologic-radiomics nomogram stratified DFS in HPV-positive OPSCC. Supports peritumoural context and shows a pathway to clinically usable risk tools.
Lang et al. [47]	2021	Retrospective; external test cohort	Evaluate segmentation-free deep learning on CT for HPV status prediction.	Three-dimensional CNN with transfer learning (video pretraining on Sports-1M/C3D); end-to-end classification without tumour contours.	External test AUROC ≈ 0.81; outperformed 2D ImageNet-pretrained and 3D CNN trained from scratch.	Multiple public TCIA datasets for training; independent external test set.	Segmentation-free 3D CNNs can classify HPV on CT at competitive performance. Reduces dependence on manual contours and suggests scalable deployment.
MD Anderson H&N QI Working Group [40]	2018	Large retrospective development + holdout testing	Derive a CT radiomic signature of local recurrence (LR) after (chemo)RT in OPC.	IBEX (MATLAB) feature extraction; machine learning feature selection; clustering/partitioning; Kaplan–Meier validation.	Two-feature signature stratified LR risk with significant KM separation across train/tune/test; AUCs not emphasised.	n = 465 OPC treated with IMRT/CRT; single institution with internal splits.	A compact CT texture signature predicted local control. Establishes prognostic value of CT radiomics beyond HPV classification for clinically relevant endpoints.
Fanizzi et al. [45]	2024	Multi-centre external test (methodological DL)	Build an explainable CNN for HPV status on CT and visualise decision regions (Grad-CAM).	Inception-V3 CNN trained on OPC-Radiomics (TCIA); Grad-CAM explainability; independent multi-centre test.	Independent test AUC 0.735; accuracy 65.2%.	Train n = 499 (TCIA); independent test n = 92 from 5 centres in Italy.	Explainable DL achieved moderate generalisation and highlighted intratumoural and edge regions as decision drivers. Progress towards transparent models that clinicians can interrogate, aiding adoption.
Kann et al. [3]	2023	Retrospective evaluation on randomised trial cohort (ECOG-ACRIN E3311)	Evaluate a CT-based deep learning model for detecting pathological extranodal extension (ENE) in HPV-associated OPC.	Three-dimensional deep learning classifier; node-level annotations; comparison vs. four expert radiologists.	ENE AUC ~0.85–0.86; at matched specificity, DL sensitivity ~75%; at 30% false-positive rate, sensitivity ~ 90%.	In total, 178 scans; 313 nodes (23% ENE overall, 13% > 1 mm ENE); multi-centre trial data.	DL outperformed expert readers for ENE detection in a de-escalation trial cohort. Supports AI-assisted nodal risk assessment for treatment selection in HPV-associated OPC.
Mackin et al. [48]	2015	Technical phantom study	Quantify how CT reconstruction kernels and acquisition affect radiomic features.	Feature extraction across phantoms; kernel/slice-thickness comparisons; statistical variability analysis.	Showed substantial kernel- and slice-thickness-dependent variability in many features.	Imaging phantoms across multiple scanners/reconstructions.	Many CT radiomic features are not directly comparable across kernels/thicknesses. Underscores the need for harmonisation and protocol control in multi-centre radiomics.
Zwanenburg et al. (IBSI) [14]	2020	Consensus/standards paper	Provide Image Biomarker Standardisation Initiative (IBSI) definitions and benchmarking.	Standardised feature nomenclature/definitions; benchmarking datasets; reporting guidelines.	—	Methodology consensus (no patient cohort).	Harmonised radiomics features and reporting recommendations. Enables reproducibility and cross-study comparability, especially critical in CT radiomics.
Fournier et al. (EIBALL/EORTC) [29]	2021	Consensus guidance	Define tiers of validation, endpoints, and reporting for radiomics/biomarker studies.	Consensus methodology; emphasis on pre-registration, calibration, external validation.	—	Methodology consensus (no patient cohort).	Practical guidance for designing and reporting imaging biomarker studies. Roadmap for moving CT radiomics and DL models towards clinical trials and adoption.

**Table 4:** FDG-PET/CT metrics and radiomics in OPSCC: summary of PET volumetrics (MTV/TLG), texture/radiomics features, modelling strategies, performance metrics, cohorts, and main outcomes for risk stratification, response assessment, and post-CRT decision-making. Entries include research from the paper and further peer-reviewed studies not covered in the narrative, expanding the evidentiary base beyond the article’s core citations.

Author	Year	Type of Study	Objectives	Software/Computational Analysis	Results (AUC/C-Index/etc.)	Cohort (n, Setting)	Main Outcomes—What They Found and Why It Matters
Lim et al. [49]	2012	Retrospective observational (treatment-uniform cohort)	Test whether baseline PET volumetric burden (MTV/TLG) predicts outcomes more robustly than SUV metrics in OPSCC.	Lesion segmentation by relative-threshold; MTV/TLG computed on pre-CRT FDG-PET/CT; multivariable Cox models.	Higher baseline MTV/TLG independently associated with inferior locoregional control, distant metastasis, PFS, and OS; SUVmax lost significance after adjustment.	n = 176 OPSCC; definitive CRT.	Volumetric burden (MTV/TLG) outperformed single-voxel SUV measures for prognostication. Established MTV/TLG as preferred PET biomarkers for baseline risk in OPSCC.
Romesser et al. [56]	2014	Retrospective observational (uniform CCRT)	Compare the prognostic value of SUVmax, GTV, and MTV in OPSCC treated with platinum-based CCRT.	Fixed- and relative-threshold segmentation; Cox regression for LRC, DM, PFS, OS.	MTV independently predicted LRC, DM, PFS, and OS; SUVmax was not independently prognostic after adjustment.	n = 100 OPSCC; single institution; uniform CCRT.	MTV consistently outperformed SUVmax for all key endpoints. Reinforces volumetric metrics as the PET class to prioritise in clinical models.
Kim et al. [50]	2015	Retrospective observational	Evaluate prognostic significance of SUVmax and MTV on pretreatment FDG-PET/CT in OPSCC.	Relative-threshold MTV; univariable and multivariable Cox models for OS/DFS.	On multivariable analysis, SUVmax and MTV were independent predictors of OS/DFS; reported cut-offs around MTV ≈ 11 mL and TLG ≈ 79 g.	n = 221 OPSCC; pre-CRT PET/CT; single centre.	Both MTV and SUVmax showed independent prognostic value, with MTV capturing overall disease burden. Confirms volumetric burden is clinically relevant and may complement SUV-based assessment.
Alluri et al. [51]	2014	Retrospective observational (HPV-positive stage III–IV)	Assess whether MTV predicts outcomes specifically in HPV-positive advanced OPSCC.	MTV of primary + nodes; Cox models adjusted for clinical covariates.	Total MTV and primary tumour MTV independently predicted survival; SUVmax/SUVmean not associated with event-free survival after adjustment.	n ≈ 70 HPV-positive stage III–IV OPSCC; single centre.	MTV retained prognostic value even within HPV-positive disease. Supports HPV-aware modelling and argues against universal thresholds.
Cheng et al. [53]	2013	Retrospective observational (advanced T-stage OPSCC)	Determine whether PET heterogeneity features add prognostic signal beyond MTV/TLG.	GLCM uniformity and volumetric burden (TLG); multivariable Cox and risk-score stratification.	Uniformity and TLG were independent predictors of PFS/DSS; high-risk group defined by TLG > 121.9 g and uniformity ≤ 0.138 had significantly worse survival.	n = 70 advanced T-stage OPSCC; pretreatment PET/CT.	A simple combined score (TLG + uniformity) stratified outcomes. Demonstrates that texture/heterogeneity provides complementary risk information to volumetric burden.
Mena et al. [52]	2017	Retrospective observational	Assess prognostic value of PET/CT texture features for recurrence and survival in OPSCC, controlling for MTV/TLG.	GLRLM/GLSZM/NGTDM features; multivariable Cox adjusted for MTV/TLG and clinicopathologic factors.	Selected heterogeneity features remained independently associated with recurrence and survival after adjusting for MTV/TLG.	Single-centre OPSCC cohort; pretreatment PET/CT.	Texture features add independent prognostic information. Supports incorporating heterogeneity metrics into PET-based risk models.
Haider et al. [2]	2020	Multi-institutional retrospective (external cohorts for OS/PFS)	Test whether PET/CT radiomics improves survival prognostication beyond AJCC 8th staging and clinical covariates.	Radiomics extraction; machine learning survival models; comparison against AJCC 8th baseline.	Radiomics models improved PFS/OS risk stratification; in HPV-associated OPSCC, best radiomics C-index ≈ 0.62 vs. AJCC ≈ 0.54; combined clinical + radiomics performed best.	PFS n = 311; OS n = 306; multi-institutional.	PET/CT radiomics added prognostic value beyond staging. Supports clinical integration as an augmentation to standard risk models.
Mehanna et al. (PET-NECK) [5]	2016	Randomised controlled trial	Compare PET-CT–guided surveillance versus planned neck dissection after CRT for advanced nodal HNSCC (HPV-positive OPSCC subset).	PERCIST-aligned response assessment; intention-to-treat analysis.	PET-guided surveillance was non-inferior for overall survival and reduced neck dissections by ~80%, with fewer complications and lower costs.	Multi-centre UK RCT; post-CRT surveillance.	PET-CT–guided follow-up safely avoided most planned neck surgeries. Practice-changing evidence supporting PET in post-CRT decision pathways.
Wahl et al. (PERCIST) [17]	2009	Methods/criteria (consensus)	Define PET Response Criteria in Solid Tumours (PERCIST 1.0).	Standardised SULpeak measurement and response categories; harmonised acquisition recommendations.	N/A (methods paper).	Consensus and illustrative datasets.	Provided a uniform framework for quantitative PET response assessment. Enables comparable longitudinal endpoints across studies and centres.
Hyun, Lodge, and Wahl (Practical PERCIST) [57]	2016	Methods/guide	Simplify and operationalise PERCIST for clinical research practice.	Standardised procedures for SULpeak, reference regions, and response thresholds.	N/A (practical guide).	Educational guide with examples.	Practical checklist and worked examples to implement PERCIST. Improves adherence and comparability in PET response studies.
HECKTOR 2022 challenge (overview) [54]	2023	Multi-centre benchmarking challenge (MICCAI)	Benchmark automatic PET/CT segmentation (GTVp/GTVn) and recurrence-free survival prediction on blind test data.	Deep learning and radiomics submissions; blind evaluation; public baselines.	Best Dice similarity (aggregate) ≈ 0.79 for segmentation; top C-index ≈ 0.68 for RFS prediction on blind test.	In total, ≈883 cases from 9 centres; harmonised preprocessing; blind test server.	Fully automated PET/CT pipelines are feasible at scale; performance varies. Provides realistic generalisability benchmarks and highlights the need for standardisation.
Andrearczyk et al. (MedIA HECKTOR) [54]	2023	Challenge summary (second edition)	Summarise PET/CT tumour segmentation and outcome-prediction findings from the 2021 HECKTOR challenge.	Comparative analysis of top DL/radiomics pipelines; blind test outcomes.	Segmentation and outcome prediction feasible; several methods achieved C-index > 0.70 for survival tasks.	A 2021 multi-centre challenge dataset; blind test.	Community benchmarks show credible performance ceilings and pitfalls. Encourages external validation and rigorous reporting for PET/CT AI.

**Table 5:** Methodological drivers of heterogeneity and minimum reporting requirements.

Domain	Methodological Choice	How It Drives Heterogeneity in Claimed Performance	Minimum Reporting Requirement	Practical Mitigation/Best Practice
DWI/ADC	b-value scheme + fitting model (monoexponential vs. IVIM/DTI); fat suppression; distortion correction	Changes absolute ADC/derived parameters and noise-floor behaviour → threshold drift; inconsistent ΔADC magnitude → divergent effect sizes/AUCs across studies	List all b-values; fitting model; voxel size; TE/TR; distortion/eddy-current correction; motion handling; scanner/vendor/field strength	Use consensus b-value sets where feasible; report model explicitly; apply distortion correction; phantom/QC; sensitivity analysis across models
DWI/ADC	Timing of on-treatment imaging (e.g., week 1–2 vs. mid-course vs. end)	ΔADC depends on timepoint and therapy kinetics → different “optimal” cut-points and predictive strength across cohorts/trials	Exact timepoint(s) (fractions/days), therapy context (RT vs. CRT), and whether images are pre/post adaptive replans	Pre-specify timepoints; align to trial decision points; report time-window; analyse timepoint interactions
DWI/ADC	Segmentation rules (primary vs. nodal; whole lesion vs. solid tumour; necrosis/cyst inclusion; peritumour)	ROI definition alters histogram/texture features → different prognostic associations and model calibration; inflates between-study variability	Segmentation target(s); necrosis/cyst handling; peritumour margin; who segmented; inter/intraobserver assessment; software	Publish segmentation protocol; use multi-reader QC; consider robustness testing across ROI variants
MRI radiomics	Preprocessing (resampling; denoising; intensity normalisation; bias-field correction)	Shifts feature distributions and stability; inconsistent preprocessing → poor transferability and heterogeneous reported AUCs	Resampling method + target spacing; normalisation strategy; any filtering/denoising; registration steps	Standardise preprocessing; report parameters; perform test–retest/perturbation robustness checks
MRI radiomics	Feature definitions/discretisation (IBSI compliance; bin width/number; filter banks)	Non-comparable feature values across pipelines → apparent disagreements in “top features” and performance	Feature set/version; IBSI compliance statement; discretisation method; software/version	Use IBSI-aligned tools; lock feature definitions; provide code/config; sensitivity analyses to binning
CT radiomics/DL	Acquisition + reconstruction (slice thickness; kernel; contrast phase; dose; metal artefact)	Strongly affects texture features; heterogeneous imaging physics → site signature; external AUC drop despite strong internal CV	Scanner/vendor; slice thickness; kernel; contrast timing; dose parameters; artefact handling/exclusions	Protocol harmonisation; stratify splits by site; report domain shift; consider physics-aware normalisation
CT radiomics/DL	Resampling, HU discretisation, intensity normalisation; tumour vs. nodal vs. peritumour features	Alters texture scale and heterogeneity metrics; peritumour adds sensitivity to contour variability → inconsistent gains across studies	Target spacing; discretisation/bin width; peritumour definition; contouring policy	Report and fix these choices; test peritumour robustness; multi-centre external validation
FDG-PET/CT	Uptake time, blood glucose, reconstruction, partial volume correction	SUV scaling differences propagate to MTV/TLG and texture; inconsistent conditions → threshold drift and varied prognostic strength	Dose, uptake time, glucose; reconstruction settings; PVC method; scanner/vendor	Standardise uptake/recon; include harmonised protocols; consider accreditation/QC; sensitivity analyses
FDG-PET/CT	MTV/TLG delineation method (fixed SUV threshold vs. %SUVmax vs. gradient/algorithmic)	MTV/TLG values depend on delineation rule → heterogeneous cut-offs and effect sizes; limits trial comparability	Exact delineation method + parameters; whether primary/nodal summed; handling of multifocal disease	Use consensus methods; report parameters; evaluate inter-method agreement; calibrate cut-offs to method
FDG-PET radiomics	Texture computation (voxel size; discretisation; grey-level quantisation; motion/respiratory blur)	Texture highly sensitive to quantisation and spatial resolution → “same feature name” yields different values/performance	Voxel size used for texture; quantisation approach; filtering; motion management	Resample to standard voxel sizes; lock discretisation; include motion mitigation where possible
Multi-centre harmonisation	Harmonisation technique (e.g., ComBat variants), covariates included, and where it is fit (train-only vs. full dataset)	Without harmonisation: site-driven variance masquerades as signal; with leakage: inflated AUC; with over-correction: true biology removed	Method + variant; covariates; whether fitted on training only; diagnostics (pre/post distributions)	Fit harmonisation within training folds; report diagnostics; consider scanner as random effect; external validation
Modelling/validation	Data splitting, feature selection and tuning (leakage), calibration, and choice of endpoint	Optimistic internal performance and unstable estimates drive “headline AUC spread” across trials; endpoint definitions further fragment results	Split strategy; leakage safeguards; nested CV; external validation; CIs; calibration; endpoint definition	Pre-register analysis; nested CV; report calibration + decision-curve where possible; validate on site-/time-separated cohorts

Summary of key methodological choices across quantitative imaging studies (e.g., acquisition parameters, segmentation rules, preprocessing/feature definitions, harmonisation strategies, and validation design) that plausibly contribute to between-study variability in reported performance. The table provides a concise checklist of minimum reporting requirements and practical mitigation strategies to support reproducible study design, multi-centre comparability, and clinical translation.

## Data Availability

No new data were created or analysed in this study. Data sharing is not applicable to this article.

## Abbreviations

1.5 T	1.5 tesla (MRI field strength)
ADASYN	Adaptive synthetic sampling
ADC	Apparent diffusion coefficient
ADCb0-1000	Apparent diffusion coefficient calculated using b = 0 and b = 1000 s/mm^2^ (ADC b0–1000)
ADCmean	Mean apparent diffusion coefficient
AI	Artificial intelligence
AJCC	American Joint Committee on Cancer
AJCC-8	AJCC 8th edition staging system
ANCOVA	Analysis of covariance
AUC	Area under the (ROC) curve
AUC-CSH	Area under the curve of the cumulative SUV–volume histogram (AUC-CSH)
AUROC	Area under the receiver operating characteristic curve
C-index	Concordance index
C3D	C3D (3D convolutional neural network architecture)
CAM	Class activation mapping
CCRT	Concurrent chemoradiotherapy
CE	Contrast-enhanced
CE-T1	Contrast-enhanced T1-weighted imaging
CE-T1/T2	Contrast-enhanced T1-weighted imaging/T2-weighted (MRI sequence)
CI	Confidence interval
CNN	Convolutional neural network
CoLlAGe	Co-occurrence of Local Anisotropic Gradient Orientations (radiomic feature family)
ComBat	ComBat harmonisation method
CRT	Chemoradiotherapy
CT	Computed tomography
CT/MRI	Computed tomography/magnetic resonance imaging
DFS	Disease-free survival
DFS/OS	Disease-free survival/overall survival
DL	Deep learning
DM	Distant metastasis
DW-MRI	Diffusion-weighted magnetic resonance imaging
DWI	Diffusion-weighted imaging
DWI/ADC	Diffusion-weighted imaging/apparent diffusion coefficient
E3311	ECOG-ACRIN E3311 trial (OPSCC de-escalation trial)
ECOG-ACRIN	ECOG-ACRIN Cancer Research Group (Eastern Cooperative Oncology Group + ACRIN)
EFS	Event-free survival
EIBALL/EORTC	EIBALL/EORTC (joint guidance)
ENE	Extranodal extension
EPI	Echo-planar imaging
FDG	(18F-)fluorodeoxyglucose
FDG-PET	(18F-)fluorodeoxyglucose positron emission tomography
FDG-PET/CT	(18F-)fluorodeoxyglucose PET/CT
FDR	False discovery rate
GLCM	Grey-level co-occurrence matrix
GLRLM	Grey-level run-length matrix
GLRLM/GLSZM/NGTDM	Grey-level run-length matrix/grey-level size zone matrix/neighbourhood grey-tone difference matrix
GLSZM	Grey-level size zone matrix
Grad-CAM	Gradient-weighted class activation mapping
GTV	Gross tumour volume
GTV-N	Gross tumour volume—nodal
GTV-P	Gross tumour volume—primary
GTVn	Gross tumour volume—nodal
GTVp	Gross tumour volume—primary
H&N	Head and neck
HECKTOR	Head and neck tumour segmentation and outcome prediction (challenge)
HNSCC	Head and neck squamous cell carcinoma
HPV	Human papillomavirus
HPV-POSITIVE	Human papillomavirus—positive
HR	Hazard ratio
IBEX	Imaging Biomarker Explorer
IBSI	Image Biomarker Standardisation Initiative
IDEAL	IDEAL Framework (Idea, Development, Exploration, Assessment, Long-Term Study)
ImageNet	ImageNet dataset
IMRT	Intensity-modulated radiotherapy
IMRT/CRT	Intensity-modulated radiotherapy/Chemoradiotherapy
Inception-V3	Inception v3 (CNN architecture)
KM	Kaplan–Meier
LR	Local recurrence
LR/RF	Logistic regression/random forest
LRC	Locoregional control
MATLAB	MATLAB (MathWorks numerical computing environment)
MICCAI	Medical Image Computing and Computer Assisted Intervention
MITK	Medical Imaging Interaction Toolkit
MR	Magnetic resonance
MRgRT	Magnetic resonance-guided radiotherapy
MRI	Magnetic resonance imaging
MRMR	Minimum redundancy maximum relevance
MTV	Metabolic tumour volume
MTV/TLG	Metabolic tumour volume/total lesion glycolysis
NAC	Neoadjuvant chemotherapy
NGTDM	Neighbourhood grey-tone difference matrix
NPC	Nasopharyngeal carcinoma
NPV	Negative predictive value
OAR	Organ at risk
OPC	Oropharyngeal cancer
OPSCC	Oropharyngeal squamous cell carcinoma
OPSCC-HPV	HPV-associated OPSCC
OS	Overall survival
OS/DFS	Overall survival/disease-free survival
OS/PFS	Overall survival/progression-free survival
PERCIST	PET Response Criteria in Solid Tumours
PET	Positron emission tomography
PET-CT	Positron emission tomography/computed tomography
PET-NECK	PET-NECK trial (PET-guided surveillance vs. neck dissection after CRT)
PET/CT	Positron emission tomography/computed tomography
PET/DWI	Positron emission tomography/diffusion-weighted imaging
PET/MRI	Positron emission tomography/magnetic resonance imaging
PET/MRI/CT	Positron emission tomography/MRI/CT
PFS	Progression-free survival
PFS/DFS	Progression-free survival/disease-free survival
PFS/DSS	Progression-free survival/disease-specific survival
PFS/DSS/OS	Progression-free survival/disease-specific survival/overall survival
PFS/OS	Progression-free survival/overall survival
PPV	Positive predictive value
QI	Quality improvement
QIBA	Quantitative Imaging Biomarkers Alliance
R-IDEAL	R-IDEAL framework for radiotherapy innovation evaluation
RCT	Randomised controlled trial
RFS	Recurrence-free survival
ROC	Receiver operating characteristic
ROC/AUC	Receiver operating characteristic/area under the (ROC) curve
ROI	Region of interest
RRS	Radiomics risk score
RT	Radiotherapy
SCC	Squamous cell carcinoma
SD	Standard deviation
SMOTE	Synthetic Minority Over-Sampling Technique
SPM12	Statistical Parametric Mapping (version 12)
SPSS	Statistical Package for the Social Sciences
SULpeak	Peak standardised uptake value (lean body mass-normalised)
SUV	Standardised uptake value
SUVmax	Maximum standardised uptake value
SUVmean	Mean standardised uptake value
SUVpeak	Peak standardised uptake value
SVM	Support vector machine
T1	T1-weighted (MRI sequence)
T1WI	T1-weighted imaging
T2	T2-weighted (MRI sequence)
T2WI	T2-weighted imaging
TCIA	The Cancer Imaging Archive
TLG	Total lesion glycolysis
UK	United Kingdom
VOI	Volume of interest
XGBoost	eXtreme Gradient Boosting
ΔADC	Change in apparent diffusion coefficient

## Appendix A

(1) PubMed/MEDLINE (searched via PubMed)—run on 10 July 2025

Filters applied in PubMed: Humans; English; publication dates 1 January 2009–1 June 2025

PubMed Search 1 (Master HPV+ OPSCC quantitative imaging)

Disease/site terms: “oropharyngeal” OR “OPSCC” OR “oropharyngeal squamous cell carcinoma” OR “head and neck squamous cell carcinoma” OR “HNSCC”

HPV terms: “HPV” OR “p16” OR “human papillomavirus”

Quantitative imaging/AI terms: “diffusion” OR “DWI” OR “ADC” OR “apparent diffusion coefficient” OR “IVIM” OR “radiomics” OR “texture analysis” OR “machine learning” OR “deep learning” OR “artificial intelligence” OR “FDG PET” OR “PET/CT” OR “CT” OR “MRI”

Fielding: Title/Abstract for keywords; MeSH where appropriate.

(In PubMed, these were combined using AND between the three concept groups, and OR within each group.)

PubMed Search 2 (DWI/ADC and longitudinal response in HPV+ OPSCC)

Disease/site terms (as above)

HPV terms (as above)

DWI/ADC terms: “diffusion-weighted” OR “DWI” OR “diffusion” OR “ADC” OR “apparent diffusion coefficient” OR “IVIM”

Longitudinal/response terms: “baseline” OR “mid-treatment” OR “intratreatment” OR “during radiotherapy” OR “longitudinal” OR “response” OR “chemoradiotherapy” OR “radiotherapy”

PubMed Search 3 (Radiomics/ML/DL across MRI/CT/PET in HPV+ OPSCC)

Disease/site terms (as above)

HPV terms (as above)

Radiomics/AI terms: “radiomics” OR “texture” OR “texture analysis” OR “machine learning” OR “deep learning” OR “convolutional neural network” OR “CNN” OR “artificial intelligence”

Imaging modality terms: “CT” OR “MRI” OR “PET” OR “FDG” OR “PET/CT”

PubMed Search 4 (PET quantitative metrics, response, surveillance)

Disease/site terms (as above)

HPV terms (as above)

PET terms: “PET” OR “FDG” OR “positron emission tomography”

Quantitative/response terms: “SUV” OR “metabolic tumor volume” OR “MTV” OR “total lesion glycolysis” OR “TLG” OR “PERCIST” OR “response criteria” OR “surveillance” OR “neck dissection”

PubMed Search 5 (MR-Linac/MR-guided and adaptive radiotherapy integration)

Disease/site terms (as above)

HPV terms (as above)

MR-Linac/adaptive terms: “MR-Linac” OR “MR guided” OR “MR-guided” OR “adaptive radiotherapy” OR “online adaptive” OR “response-adaptive”

Functional imaging terms: “diffusion” OR “DWI” OR “ADC” OR “functional imaging”

PubMed Search 6 (Methodology/translation: standardisation, harmonisation, external validation)

(Broader head & neck focus; HPV restriction not required to capture standards/harmonisation papers.)

Disease/site terms: “head and neck” OR “HNSCC” OR “oropharyngeal” OR “OPSCC”

Methods terms: “standardisation” OR “standardization” OR “reproducibility” OR “repeatability” OR “harmonisation” OR “harmonization” OR “ComBat” OR “multicentre” OR “multicenter” OR “external validation” OR “calibration” OR “quality score” OR “IBSI”

Imaging terms: “radiomics” OR “diffusion” OR “ADC” OR “PET” OR “FDG”
